# Editorial: Novel Developmental Perspectives on the Link Between Morality and Social Outcomes

**DOI:** 10.3389/fpsyg.2022.888373

**Published:** 2022-03-30

**Authors:** Simona C. S. Caravita, Miriam H. Beauchamp, Robert Thornberg

**Affiliations:** ^1^Norwegian Centre for Learning Environment and Behavioural Research in Education, University of Stavanger, Stavanger, Norway; ^2^Department of Psychology, Catholic University of the Sacred Heart, Milan, Italy; ^3^Department of Psychology, University of Montreal, Montreal, QC, Canada; ^4^Sainte-Justine Hospital Research Center, Montreal, QC, Canada; ^5^Department of Behavioural Sciences, University of Linköping, Linköping, Sweden

**Keywords:** morality, moral development, social behavior, socialization, neuroscience, novel perspectives

Morality is a complex construct examined in research from a number of disciplinary perspectives. Often thought of as the ability *to decide* about rightness or wrongness in situations involving a person's well-being and in terms of concerns about justice, rights, caring and virtues, morality also refers to the ability *to regulate behaviors* affecting others. Although the constructs overlap, moral cognition, moral standards and moral actions (social outcomes) are not equivalent; their relation and gaps can be altered by emotions and social influences. The complex association between moral standards and actions has been investigated in psychological and educational sciences, neurosciences, and philosophy, often separately and from different angles. From a developmental point of view, some researchers (e.g., Bandura, 1986) emphasized the possibility that moral cognition and action originate from social learning and transactions within social contexts, while others (e.g., Haidt, 2001) suggested that morality stems primarily from human biological organization.

In recent years, the scientific debate on the roots of morality and the relationship between moral cognition and behavior has produced different, sometimes contradictory, theorizations and studies with equivocal results. Difficulties in disentangling the origins of morality and capturing to what extent moral cognition and standards translate into social behavior also reflects the complexity of developing assessment measures able to accurately quantify or qualify moral processes in relation to social dimensions and outcomes. This Research Topic aims to contribute to this interdisciplinary scientific debate about morality with nine papers providing novel contributions in terms of theorization, empirical data and the development of new measures, and focusing on the developmental span.

With regards to the origins of morality, in their theoretical contribution, Carpendale et al. argue in favor of overcoming a Cartesian-split-mechanistic view of morality as originating from culture or biology. They propose a novel process-relational perspective about knowledge and morality as constructed through social interaction and as a process of coordinating perspectives. Four other papers deepen the debate about the role played by moral processes related to self-justifying one's own transgressions (*moral disengagement*; Bandura, 1986) in the social phenomenon of bullying. The literature has provided consistent evidence that the proneness to morally disengage is linked with higher levels of bullying behaviors and lower proneness to defend bullied peers (e.g., Thornberg et al., 2015). Nevertheless, there is still debate about the extent to which this type of moral cognition is correlated with being a passive bystander in bullying, about the interplay of moral disengagement mechanisms and other moral dimensions in the explanation of behaviors in bullying, and about the role played by moral disengagement in relation to bias-based bullying (e.g., ethnic bullying). The four papers provide novel contributions to this debate. By means of longitudinal data, Falla et al. shed further light on the complex associations that exist between social behaviors and morality, providing evidence that perpetrating negative behaviors (bullying) can increase moral disengagement and decrease empathy, and that some moral disengagement mechanisms mediate the link between behavior and empathy. Caravita et al. for their part, contribute to clarifying that moral disengagement and other forms of moral cognition (comprehension of rules) are separate mechanisms, and when they are both taken into account, moral disengagement is the only moral cognitive dimension associated with the perpetration of bullying. Further highlighting the complexity of moral mechanisms and their associations with social outcomes, Iannello et al. show that moral disengagement mechanisms mediate the association between ethnic prejudice and perpetrating ethnic bullying. They also provide novel results suggesting that closeness to teachers (an emotional contextual factor) can help restrain morally disengaged children from perpetrating bullying. The study by Lo Cricchio et al. provides a novel measure to assess moral disengagement in situations of ethnic bullying, thus presenting moral disengagement in a situational perspective. These studies offer important insights also for a moral component in anti-bullying intervention.

At the crossroads of neuroscience and developmental psychology research, Bacchini et al. contribute to the limited literature on deontological vs. utilitarian moral reasoning in adolescence (e.g., Caravita et al., 2017) with an innovative study showing how these forms of moral reasoning are related to individual factors (callous-unemotional traits and moral disengagement proneness) and contextual experiences (perceived parental rejection and exposure to community violence). A further novel contribution to the research on the development of moral cognition comes from the study by Zhou and Wong, who compare the understanding of restorative vs. retributive justice among children between 5 and 8 years of age. They find that a higher preference for a restorative justice approach emerges with age.

The relevance of positive life experiences and relationships in early childhood to build positive socio-moral temperament is investigated in a cross-cultural study presented in the article by Narvaez et al. Results highlight the relevance of guaranteeing children's emotional wellbeing, in terms of happiness and thriving and low depression and anxiety, to promote their self-regulation and positive moral socialization outcomes through socio-moral temperament. Lastly, a novel methodological contribution to research on moral reasoning comes from the study by Zarglayoun et al. who developed the MorALERT serious videogame to assess and strengthen moral reasoning skills and competencies. This tool offers new possibilities for assessment, remediation and intervention research in this area.

Together, the nine papers enrich knowledge on moral processes, their development and how they are linked to social behaviors, and open new important avenues and lines of research on this topic.

## Author Contributions

All authors listed have made a substantial, direct, and intellectual contribution to the work and approved it for publication.

## Conflict of Interest

The authors declare that the research was conducted in the absence of any commercial or financial relationships that could be construed as a potential conflict of interest.

## Publisher's Note

All claims expressed in this article are solely those of the authors and do not necessarily represent those of their affiliated organizations, or those of the publisher, the editors and the reviewers. Any product that may be evaluated in this article, or claim that may be made by its manufacturer, is not guaranteed or endorsed by the publisher.
